# How community resources mitigate the association between household poverty and the incidence of adverse childhood experiences

**DOI:** 10.1007/s00038-019-01258-5

**Published:** 2019-05-28

**Authors:** Alexandra Blair, Louise Marryat, John Frank

**Affiliations:** 1 Département de médecine sociale et préventive, École de santé publique, Université de Montréal, Montreal, Canada; 2 Centre de recherche du Centre hospitalier de l’Université de Montréal, 850 Ave. St-Denis, #S03-710, Montreal, Quebec Canada; 3 Scottish Collaboration for Public Health Research and Policy, University of Edinburgh, Edinburgh, UK; 4 The Farr Institute of Health Informatics Research, Edinburgh, UK; 5 Salvesen Mindroom Research Centre, Centre for Clinical Brain Sciences, University of Edinburgh, Edinburgh, UK; 6 Usher Institute of Population Health Sciences and Informatics, University of Edinburgh, Edinburgh, UK

**Keywords:** Childhood circumstances, Child health, Adverse childhood experiences, Poverty, Inequalities, Health equity, Social epidemiology

## Abstract

**Objectives:**

To assess what proportion of the association between household low income and incidence of adverse childhood experiences (ACE) would be eliminated if all households had access to housing, transportation and childcare services, breastfeeding counselling, and parks.

**Methods:**

Using Growing Up in Scotland birth cohort data (*N* = 2816), an inverse probability-weighted regression-based mediation technique was applied to assess associations between low-income status (< £11,000 in 2004/5), resource access, and cumulative 8-year ACE incidence (≥ 1, ≥ 3 ACEs). Resource access was measured based on households’ self-reported difficulties (yes/no) in accessing housing, transportation, childcare, and breastfeeding counselling, and park proximity (within 10 min from the residence).

**Results:**

The protective effects of resources were heterogeneous. Only access to transportation was associated with lower ACE incidence in both low- and higher-income households. If all had access to transportation, 21% (95% CI 3%, 41%) of the income-based inequality in incidence of 3 or more ACEs could be eliminated.

**Conclusions:**

While second best to the elimination of child poverty, measures to improve families’ access to community resources such as transportation may mitigate the effects of poverty on ACE incidence.

**Electronic supplementary material:**

The online version of this article (10.1007/s00038-019-01258-5) contains supplementary material, which is available to authorized users.

## Introduction

Adverse childhood experiences (ACEs), which include forms of abuse, neglect, and household dysfunction (Felitti et al. 1998), have been shown to influence academic achievement, health behaviours (e.g. smoking, alcohol, and drug use), and various health conditions (e.g. diabetes, depression, heart disease) throughout the life course (Cambois and Jusot 2011; Nandi et al. 2012). Setting the stage for lifelong health and social outcomes, (Nurius et al. 2015) ACEs merit attention both from the moral perspectives of child welfare and health justice (Venkatapuram 2013), and from perspectives of life course health promotion and societal prosperity (Schady 2015).

On average, children living in low-income households tend to experience a greater number of ACEs than their higher-income peers (Wade et al. 2014). In Scotland, where ACE prevalence is high [i.e. 65% of children experience one or more ACEs by the age of 8 years (Marryat and Frank 2019)], 53% of children in the highest income households are ACE-free by age eight, compared to 8% in the lowest income households (Marryat and Frank 2019). These inequities beg the question of whether the relationship between low-income and ACE incidence can be mitigated. A growing body of theoretical and empirical work suggests that the experience of low-income, and its association with health outcomes, can vary according to the relative generosity of state investment in benefits, social policies, and resources. For example, the generosity of unemployment benefits is known to influence psychological distress (O’Campo et al. 2015) and self-reported health (Bambra and Eikemo 2009) among the unemployed. It is possible, therefore, that the association between low-income and child adversity could be mitigated by protective social, economic, or infrastructural resources.

The assumed modifiability of poverty experience—in terms of its impact on childhood adversity—underpins many of the UK and Scotland’s policy recommendations for investment in early childhood development, as a means of reducing health inequities across the life course. Reports published between 1980 and 2014 (Acheson 1988; Black et al. 1980; Marmot et al. 2010; Scottish Government 2014) all discuss promoting access to adequate and affordable housing, transportation, childcare services, and recreational opportunities to ensure children are assured the “best start in life” (Scottish Government 2014). These features of local environments have been identified as upstream causes of health inequalities, and interventions on the latter domains are believed to be among the most effective at reducing life course health inequities (Smith and Kandlik Eltanani 2015).

The objectives of this study were therefore to assess whether certain community resources (namely, self-reported access to housing, local public parks or play parks, childcare services, transportation services, or formal, in-person pre- or perinatal counselling—specifically for breastfeeding) are associated with lower ACE incidence in households above and below the poverty line and to assess the extent to which income inequalities in 8-year cumulative ACE incidence could be eliminated if all had access to these community resources.

## Methods

This study used data from the Growing Up in Scotland (GUS) birth cohort, which followed children born in 2004/2005 (*N* = 5217 recruited at age 10 months) yearly, for seven sweeps of data collection, until 2013/2014 (age 7–8 years) (response was 66% by Sweep 7, *N* = 3456) (Anderson et al. 2007). With a sampling frame based on Scotland’s universal Child Benefit—described in detail previously (Anderson et al. 2007)—GUS documents the health, behavioural, and social characteristics of a nationally-representative sample of Scottish children. A total of 2816 children who had participated in all seven sweeps of data collection and for whom relevant ACE, income, and covariate data were available were included in this study.

### Measures

#### Outcome measure: adverse childhood experience (ACE) incidence

In GUS, data were available to measure seven experiences that are typically counted when assessing the burden of adverse childhood experiences (ACEs) (Felitti et al. 1998): child physical abuse (i.e. being pushed, grabbed, or hit) or emotional neglect (i.e. never played with, never asked about their well-being or day at school); mother’s experience of domestic violence (i.e. being pushed, held down, kicked, bitten, hit, strangled or smothered, or hurt with a weapon); parents’ reported use of street drugs or elevate use of alcohol [≥ 14 units per week (Brown et al. 2015)]; separation, divorce, or incarceration; and elevated affective symptoms in the past week [i.e. a score of 36 or higher on the SF-12’s mental health component (Gill et al. 2007), or of 1 standard deviation above the mean (z-score ≥ 1) on a restricted Depression, Anxiety and Stress Depression scale (Parkitny and McAuley 2010)]. Each ACE is described in detail in the Supplement’s eMethod 1. Since ACE-related items were asked inconsistently through the study cycles, we measured 8-year cumulative ACE incidence by summing the ACEs present for each child throughout the study period, as has been done previously (Marryat and Frank 2019).

Two incidence cut-offs were applied. First, a cut-off of zero versus one or more ACEs was used to capture both a relevant, if ambitious, objective for child welfare policy, and because children with one or more ACEs—although at lower risk for future social and health issues than their high-exposure peers (Hughes et al. 2017)—will likely experience a larger share of the population burden of future outcomes simply because they are more numerous (Rose 1992). Second, a higher cut-off was used (≥ 3 ACEs) to reflect findings that multiple, concurrent adversities place children at higher risk of negative health and social outcomes throughout the life course (Hughes et al. 2017). The three ACE cut-offs were used in lieu of a higher cut-off (e.g. ≥ 4) due to the smaller number of ACEs measured in this study (i.e. seven compared to ten), and the limited number of respondents reporting three or more ACEs by the age of 8 years.

#### Exposure measure: low income

Total household income after tax and other mandatory deductions, in pounds sterling (£), was measured at baseline and equivalized according to the number of household members based on their age (Anderson et al. 2007). To allow for the application of a weighting-based mediation analysis approach, a dichotomized measure of income was used. Household income was dichotomized according to the UK’s low-income cut-off value in 2004/2005, of £11,000 per year (Brewer et al. 2006).

#### Mediator measures: access to community resources

Five community resources were considered as potential mediators and modifiers of the association between low-income and ACE incidence. These included self-reported access to: non-precarious housing, a local park or play park, transportation services, childcare services, and formal in-person breastfeeding counselling. These factors were selected both because they had been acknowledged by governments of Scotland and the UK as relevant areas of intervention for the promotion of child health and well-being (Acheson 1988; Black et al. 1980; Marmot et al. 2010; Scottish Government 2014), and because the GUS questionnaire included sufficient items to operationalize each measure. Factors were considered separately (rather than as a summary score) both because of the potential heterogeneity in protective pathways they represent, and to facilitate interpretation. All were measured at baseline and were operationalized as dichotomous measures (absent vs. present) to allow the application of weighting-based mediation analysis approach.

Households were considered to have access to non-precarious housing if, when asked “In the first 3 months [or at the time of the survey], was there anything […] you found particularly difficult?” respondents did not identify “Accommodation or housing problems” as something that the household found particularly difficult and—following the UK’s Office for National Statistic definitions of deprived housing (2014)—did not report an absence of central heating, nor residence in a shared dwelling (i.e. renting a room or rooms within a home), a caravan, mobile home, or houseboat when asked about their current housing situation. Households reporting any of the latter experiences were considered to not have access to non-precarious housing.

The measure of access to a local park was based on responses to the question “Is there a public park or play park within 10 min walk of here [your residence]?” Those who reported “Yes” were considered to have access to a local park, while those reporting “No” were not. Park-related analyses were restricted to those living in urban settings.

Households were considered to have access to transportation services if when asked “In the first 3 months [or in the last 3 months] how much of a problem was lack of suitable transport?” they answered, “Not a problem.” Those who responded “Bit of a problem” or “A big problem” to the latter question, as well as those who listed “I would have transport difficulties getting to a provider” as the main reason they were not accessing childcare for the child were considered to not have access to suitable transportation services.

For the measure of access to in-person breastfeeding counselling, mothers were considered to have access to the latter resource if they responded “Yes” to the question “Did you receive any help or advice about breastfeeding at the time of the child’s birth?” and indicated that the source of the advice was a midwife, health visitor, a professional from the National Childbirth Trust or another voluntary group or organization, or another health professional. Those who reported “No” to the question and those who indicated that the source of the advice was from books, magazines, leaflets, friends, or family members were categorized as not having access to formal in-person breastfeeding counselling.

Lastly, for the measure of access to childcare services, respondents who answered “Yes” to the question “Do you currently get help with childcare for child on a regular basis?” and answered “No” when asked “If it was available and you could afford it, would you use a different kind of childcare provider as your main childcare provider for child [e.g. childminder, a Local Authority playgroup, preschool, creche or nursery, a community playgroup or preschool, a workplace creche or nursery, a Family Centre, or a child-carer]?” were considered to have access to childcare services. Those who responded that they did not get help with childcare on a regular basis, or were receiving help but answered that if alternative affordable childcare services were available, that they would use them, were considered to not have access to childcare services.

#### Covariates

Analyses were conditioned on factors that were assumed to be confounders of the associations between low income, the five community resources, and cumulative ACE incidence (Supplement’s eFigure 1). Covariates included mother’s or stepmother’s ethnicity (visible minority vs. white), age at birth of first child (below vs. 20 years and above), employment during pregnancy (ever employed vs. not employed), area of residence (urban vs. rural), level of education (less than Higher Grade/High School certification vs. Higher certification or above), and the child’s sex (which was assumed to be causally unassociated with low income and resource access, but was included to ensure consistency with the extant literature).

### Analyses

Descriptive analyses were performed on the sample’s characteristics (including ACE incidence according to income and resource access). We used income-stratified identity-link Poisson regression models to assess the associations between resources and ACE incidence across income groups. These models were weighted using the product of individuals’ inverse probability weight (IPW) for low-income exposure (given covariate information) and GUS longitudinal weight—which accounted for selection at baseline and non-response until the final survey sweep (Anderson et al. 2007). These IPW ensured that income groups were balanced in terms of the measured covariates. Details on the construction of IPW are described in the Supplement’s eMethods 2. Then, to assess the extent to which income inequalities in ACE incidence could be eliminated if all had access to any of the five resources, we estimated a metric known as the “Proportion Eliminated” (PE) in the mediation literature (VanderWeele 2015). The PE is estimated by first taking the difference between the total association between low income and ACE incidence (hereafter referred to as the “total effect” or “TE”), and the association between low income and ACE incidence that would remain if all had the mediating resource (hereafter referred to as the “controlled direct effect” or “CDE”); this difference is then divided by the TE [i.e. PE = (TE − CDE)/TE] (VanderWeele 2013).

For TE and CDE estimation, we used an IPW-weighted identity-link Poisson regression-based approach—the theory behind which has been described previously (VanderWeele 2009). For the TE estimate, we regressed ACE incidence on income—weighting the model using the product of individuals’ IPW for low-income exposure and GUS longitudinal weight. For the CDE estimate, we regressed ACE incidence on income, resource absence, and the product between the latter (to account for potential effect modification). The latter model was weighted using the three-way product of individuals’ IPWs for low-income exposure and for resource absence, and GUS longitudinal weight. The coefficient associated with low-income and resource presence captures the income inequality in ACE incidence that remains if all had access to the resource (the CDE) (VanderWeele 2009). All confidence intervals for the TE, CDE, and PE were estimated using the bootstrap method (500 iterations) (VanderWeele 2015).

Additionally, three sensitivity analyses were conducted. First, to assess whether the associations between low-income and the incidence of each ACE were relatively homogeneous, we specified IPW-weighted models for the incidence of each ACE, separately. Second, we applied VanderWeele’s method to assess how large the associations would have to be between an unmeasured factor and both the resource and ACE incidence for the true CDE to be null, despite non-null estimates (VanderWeele 2016). Lastly, since the use of IPW relies on the assumption of practical positivity [i.e. that propensity scores used to estimate IPW are neither 0 nor 1 (0% or 100% probability)], we performed descriptive analyses of all estimated propensity scores (VanderWeele 2015). All analyses were completed using R, version 3.4.1 (R-CoreTeam 2017).

## Results

### Baseline characteristics

Overall, a greater proportion of mothers with the following characteristics were part of low-income households: those who were not white; had not completed Highers (basic secondary school); had not been working at any point during their pregnancy; had their first child before the age of 20 years; lived in urban areas; had not been offered breastfeeding counselling; and had difficulties accessing transportation or childcare services (Table 1).

**Table 1:** Bivariate analyses of the characteristics of the Growing Up in Scotland birth cohort study sample, followed from 10 months to 8 years (2004/2005–2013/2014, *N* = 2816)

Measures	Overall (%)	Poverty line (£11,000/year)		0 ACEs^b^ by age 8 years (%)	< 3 ACEs^b^ by age 8 years (%)
		Above (%) *N* = 2232	Below (%) *N* = 584		
Overall	1.00	79	21	43	91
Household income					
Below poverty line (< £11,000)	21	0	100	12***	76***
Above poverty line (≥ £11,000)	79	100	0	47***	95***
Sex of child					
Female	48	78*	22*	46**	93***
Male	52	81*	19*	40**	90***
Mother’s age at birth of child					
≥ 20 years	82	84***	16***	46***	96***
< 20 years	18	37***	63***	17***	73***
Mother’s ethnicity					
White	97	80***	20***	43	91
Visible minority	3	59***	41***	45	91
Mother’s educational attainment					
< Highers	72	86***	14***	47***	82***
Highers, vocational, or degree certificate	28	51***	49***	24***	94***
Employment during pregnancy					
Ever employed	74	87***	14***	46***	93***
Not employed	26	52***	48***	31***	84***
Area of residence					
Urban	79	78***	22***	41***	91**
Rural	21	83***	17***	48***	94**
Absence of housing deprivation					
Yes	86	82***	18***	44***	92***
No	14	63***	38***	32***	87***
Proximity to park^a^					
Yes	92	67	33	34	86
No	8	65	35	36	89
Perceived adequate access to transportation					
Yes	80	83***	17***	46***	93***
No	20	60***	40***	27***	83***
Breastfeeding education					
Yes	74	81***	19***	45***	92**
No	26	74***	27***	36***	89**
Perceive adequate access to childcare					
Yes	59	85***	15***	45**	92
No	41	71***	29***	40**	90

*Indicates a *p* value Chi square statistic of less than 0.05

**Indicates *p* value of Chi square statistic of less than 0.01

***Indicates *p* value of Chi square statistic of less than 0.001

^a^Data are restricted to those living in urban settings (*n* = 2104)

^b^“ACEs” refer to adverse childhood experiences

### Cumulative ACE incidence

By 8 years, approximately 57% of children in the sample had experienced at least one ACE, and 9% had experienced three or more ACEs (Table 1). The proportion of children who had experienced no ACEs was 12% among those living in households below the poverty line, and 47% in households above the poverty line (Table 1). Significant associations between low income and each of the adverse childhood experiences were observed (Supplement’s eTable 1). Overall, cumulative ACE incidence (≥ 1, ≥ 3 ACEs) was lower among those who reported having access to housing, transportation, and breastfeeding counselling (Table 1, eTable 2).

### Protective role of community resources above and below the poverty line

Above the poverty line, access to housing, transportation, and breastfeeding education was associated with lower ACE incidence (Table 2). The latter resources were associated with a 10- (95% CI 6, 14), 14- (95% CI 9, 19) and 9-percentage point (95% CI 4, 13) lower incidence of 1 or more ACEs, respectively, and much smaller coefficients for three or more ACEs (RDs < 4%) (Table 2). Below the poverty line, only transportation was associated with lower ACE incidence (11- [95% CI 2, 20] and 8-percentage point [95% CI 3, 13] lower incidence of 1, and 3 or more ACEs, respectively) (Table 2).

**Table 2:** Inverse probability-weighted cumulative incidence and risk differences (RD) of 1 or more adverse childhood experiences (ACEs), or of 3 or more ACEs by age 8 years in households above and below the poverty line in the Growing Up in Scotland birth cohort (followed from 10 months to 8 years, 2004/2005–2013/2014, *N* = 2816), according to access to housing, public parks or play parks, transportation, breastfeeding education, and childcare

	Proportion with 1 more ACEs by age 8 years (%)		Proportion with 3 or more ACEs by age 8 years (%)	
	Below poverty line *N* = 584	Above poverty line *N* = 2232	Below poverty line *N* = 584	Above poverty line *N* = 2232
Housing				
Yes	82.8%	49.5%	25.3%	4.2%
No (reference)	82.3%	59.5%	24.0%	7.5%
RD% (95% CI)^a^	0.5 (− 7.8, 8.9)	− 10.0 (− 14.4, − 5.7)	1.4 (− 3.2, 5.9)	− 3.3 (− 4.7, − 1.9)
Park proximity^b^				
Yes	86.3%	52.2%	28.1%	4.9%
No	88.3%	51.1%	24.1%	3.4%
RD% (95% CI)^a^	− 2.1 (− 12.1, 7.8)	1.0 (− 4.1, 6.2)	4.0 (− 1.3, 9.5)	1.5 (0.0, 3.0)
Transportation				
Yes	80.0%	48.8%	21.8%	4.2%
No	91.0%	62.8%	29.8%	8.1%
RD% (95% CI)^a^	− 11.0 (− 19.9, − 2.2)	− 13.9 (− 18.6, − 9.3)	− 8.0 (− 12.9, − 3.2)	− 3.9 (− 5.5, − 2.4)
Breastfeeding education				
Yes	80.6%	48.6%	22.3%	4.6%
No	87.8%	57.1%	25.9%	6.1%
RD% (95% CI)^a^	− 7.2 (− 15.9, 1.4)	− 8.5 (− 13.0, − 3.9)	− 3.7 (− 8.0, 1.0)	− 1.6 (− 3.0, − 0.1)
Childcare				
Yes	86.1%	48.7%	26.0%	4.8%
No	82.1%	51.4%	21.5%	4.4%
RD% (95% CI)^a^	3.9 (− 4.6, 12.4)	− 2.6 (− 6.9, 1.7)	4.5 (0.0, 9.0)	0.4 (− 1.0, 1.7)

*RD* cumulative incidence risk difference, expressed as percentage-point (%) difference; *CI* confidence interval

^a^Cumulative incidence risk differences (RD) are weighted for mother or stepmother’s ethnicity, age at birth of the child, employment status at during pregnancy, area of residence, level of educational attainment, and child’s sex

^b^Data are restricted to those living in urban settings

### Proportion of income-based ACE inequality eliminated

TE, CDE, and PE estimates for each resource are summarized in Table 3 (full model output summarized in eTable 3 and eTable 4). With the higher ACE cut-off (≥ 3), only transportation was associated with a statistically significant PE value. If all families had access to transportation, 21% (95% CI 3%, 41%) of the income inequality in higher ACE incidence could be eliminated (Table 3). With the lower ACE cut-off (≥ 1), all PE confidence bounds crossed the null value, except those for housing (Table 3). The PE pertaining to housing was negative (PE = − 7%, 95% CI − 17%, − 0.3%), indicating that if all had access to housing, the income-based inequality in cumulative incidence of 1 or more ACEs may in fact increase. This finding is in line with findings from the above-stratified analyses: housing is associated with a 10-percentage-point lower incidence of 1 or more ACEs among wealthier households and a null association among low-income households (RD = 0.5%, 95% CI − 8%, 9%) (Table 2).

**Table 3:** Proportion of the income-based inequality in 8-year cumulative adverse childhood experience (ACE) incidence (1 or more ACEs, 3 or more ACEs) that would be eliminated if all had identified resources, in the Growing Up in Scotland Cohort (followed from 10-months to 8 years, 2004/2005–2013/2014, *N* = 2816)

Mediating resources	Total effect (TE) RD% (95% CI)^a^	Controlled direct effect (CDE) RD% (95% CI)^a^	Proportion eliminated [TE − CDE]/[TE] % (95% CI)
1 or more ACEs			
Housing	33.5 (30.6, 39.3)	37.1 (32.1, 41.6)	− 6.8 (− 16.6, − 0.3)
Park proximity^b^	34.7 (29.1, 39.7)	33.9 (29.2, 39.6)	2.1 (− 2.1, 6.6)
Transportation	33.5 (30.3, 39.0)	32.8 (27.2, 37.9)	2.1 (− 4.9, 10.0)
Breastfeeding Ed.^c^	35.0 (30.8, 39.4)	35.6 (30.4, 40.3)	− 1.4 (− 8.6, 5.7)
Childcare	32.2 (27.5, 36.7)	37.0 (30.7, 43.3)	− 14.7 (− 32.0, 3.5)
3 or more ACEs			
Housing	22.7 (17.7, 27.3)	22.9 (17.4, 28.7)	− 0.8 (− 15.0, 13.0)
Park proximity^b^	25.9 (22.2, 33.5)	26.2 (22.6, 33.9)	− 1.0 (− 8.2, 5.5)
Transportation	22.7 (18.1, 27.5)	18.0 (12.8, 23.9)	20.8 (3.4, 41.0)
Breastfeeding Ed.^c^	22.3 (17.5, 27.4)	20.6 (15.2, 26.3)	7.5 (− 7.8, 24.0)
Childcare	22.9 (17.7, 28.0)	27.5 (18.7, 36.4)	− 20.0 (− 43.5, 10.4)

*RD* cumulative incidence risk differences, expressed as percentage-point (%) difference; *CI* confidence Interval, *TE* total effect, *CDE* controlled direct effect

^a^RDs are weighted for mother or stepmother’s ethnicity, age at birth of the child, employment status at during pregnancy, area of residence, level of educational attainment, and child’s sex

^b^Park-related analyses were restricted to those living in urban settings

^c^Ed. refers to in-person education

### Sensitivity analyses

Analyses suggest that the associations between an unmeasured factor and both transportation access and ACE incidence (≥ 3 ACEs), and between an unmeasured factor and both housing and ACE incidence (≥ 1 ACEs), would have to be of RR = 3.0 (respectively) to explain away the observed CDE estimates (eTable 5). RR = 3.0 is larger than the observed association between income and incidence of either 1 or 3 or more ACEs (RR = 1.90 and RR = 1.80, respectively; data not in table). Lastly, analyses of propensity scores indicate strong covariate balance after weighting, and of practical positivity for both exposure to low income and exposure to the resources measured (eTable 6, eTable 7).

## Discussion

In a population-based sample of Scottish children, we found that access to housing, transportation, and breastfeeding education were associated with lower ACE incidence among households above the poverty line. Below the poverty line, only transportation access was associated with lower ACE incidence. We estimated that if access to transportation was held fixed across the entire population, approximately 21% of the income-based inequality in cumulative incidence of 3 or more ACEs could be eliminated.

Our finding of the protective role of transportation is in line with those of previous studies. Transportation resources can mitigate the association between distance to health or social services and service utilization (Whetten et al. 2006) and enable families in accessing employment, food, and leisure facilities (Markovich and Lucas 2011). That access to housing and breastfeeding counselling was also associated with lower ACE incidence—at least among higher-income households—is also consistent with observations in the extant literature. Housing is known to influence general family well-being by shaping residents’ mental health, sense of self-worth, and offering a stable environment from which to pursue training, employment, and parenting responsibilities (Bratt 2002). Breastfeeding resources may represent a proxy for the density of other protective health resources, such as access to nurses. Perinatal nurse visits are known to be beneficial for mothers’ health and well-being (Olds et al. 2002).

Several theories may explain the observed association between transportation and lower ACE incidence. Transportation may help parents’ to gain a sense of control over their lives—offering opportunities for decisional latitude regarding work and daily activities (Syme 1996). A second theory is that inadequate transportation is itself a stressor for low-income families. Walking as a compulsory mode of transport in disadvantaged areas is often accompanied by physical fatigue and psychosocial stress (Bostock 2001), which may lead to greater household dysfunction. Third, transportation resources allow families to access a wealth of protective resources beyond their local area (Bostock 2001). Lastly, it may be that transportation resources enable parents—specifically mothers—to remove themselves and their children from adverse situations, thus protecting their children from ACEs (Bambra 2007). These theorized mechanisms merit attention in future work.

In contrast, there are several potential explanations why statistically significant associations between parks, childcare, and ACE incidence were not observed. First, it may be that park proximity does not guarantee park use or quality (i.e. aesthetics, safety, vegetation, and equipment availability). Parks in low-income areas in the UK tend to be of lesser quality than those in higher-income areas (Fairburn et al. 2005). Similarly, childcare may not be protective if its quality is low [a well-documented issue for poor Scottish families (Siraj and Kingston 2015)] or if the parents cannot afford a critical “dose” of childcare [i.e. several half-days weekly from ages 2–4 years (Geddes et al. 2011)]. Second, it may be that these two factors play a more important protective role later in the child’s life than at the time at which they were measured (i.e. within the first year of life). Future studies on these more nuanced elements are recommended.

This study requires replication in other settings before strong recommendations can be made. If other studies confirm protective effects of similar service features, specifically for ACE prevention, intervening on community-level resource availability could represent a valuable interim measure to mitigate the association between childhood poverty on ACEs. While obviously second best to the complete elimination of poverty, local measures to promote resources for families may hold promise in settings lacking the political will for formal wealth redistribution.

These study’s findings are bound by certain limitations. First, baseline marital status was not included as a covariate (although separation is included as an ACE) due to its collinearity with the dichotomized exposure measure of household income. Approximately, 80% of single mothers were below the poverty line. This exclusion and other unmeasured factors may have contributed to residual confounding in the study. Factors that were not included in the study, primarily due to limitations in data availability, were paternal characteristics, parents’ own histories of adverse childhood experiences (i.e. as an indication of generational trauma) or behaviours (e.g. life course use of substances, incarceration, relationship status, etc.). Though sensitivity analyses suggest that the present study’s estimates are likely robust to unmeasured confounding, we recommend that, if available, future studies adopt a life course exposure perspective and consider these additional sources of bias as well as potential time-varying confounding. A second limitation pertains to the ACEs measured. Though we attempted to maximize the comparability of the ACEs measured in this study with those of previous studies (Felitti et al. 1998), missing in this study are ACEs of material neglect, sexual abuse, and emotional abuse (Felitti et al. 1998), as well as ACEs that might occur after age 8. These exclusions may lead to potential underestimation of childhood ACE incidence in the Scottish population. ACE incidence estimates may also be affected by higher observed attrition rates in the GUS cohort among younger mothers and households living in deprived areas (Marryat and Frank 2019). In this study, we sought to minimize this potential source of bias using the GUS longitudinal survey weights (Anderson et al. 2007). Furthermore, though available ACE measures were compiled here to form one single outcome measure of cumulative ACE incidence—in order to both accommodate data availability and account for the observation of the detrimental impact of concurrent adversities on health and social outcomes (Hughes et al. 2017)—this outcome operationalization can also lead to the interpretation of the moral or sociopolitical equivalency of each experience. The potential stigmatizing effect of this grouping of experiences is a limitation of both this study and its predecessors and may be a relevant topic of future enquiry. A third limitation is the potential measurement bias associated with self-reported data. Previous studies have noted differential self-reporting of neighbourhood characteristics across socioeconomic groups, wherein populations living in more deprived contexts under-report deprivation experiences, in part out of a need for self-preservation (Kawachi and Berkman 2003). It is possible, therefore, that income inequalities in resource access may have been underestimated in this study. To address these concerns, future studies may benefit from using multi-item indices to measure resource access (Echeverria et al. 2004). Lastly, the IPW-weighted models used rely on several operational assumptions. Where possible, we attempted to assess the potential sensitivity of findings to violations of these assumptions. Importantly, our findings appear robust to potential unmeasured confounding, and violations of practical positivity.

In conclusion, this is, to our knowledge, the first study to explore how community resources may mitigate the association between household poverty and cumulative ACE incidence. Of the resources assessed, only transportation was associated with lower ACE incidence in households above and below the poverty line. We estimated that a substantial portion (21%) of the income-based inequality in the 8-year cumulative incidence of three or more ACEs could be eliminated if all had access to adequate transportation. Though our findings require replication, they offer an initial body of evidence that can inform interventions to prevent both ACE incidence and income disparities in ACE incidence.

## Electronic supplementary material

Below is the link to the electronic supplementary material.

Supplementary material 1 (DOCX 267 kb)
