# Supplementary material for: How community resources mitigate the association between household poverty and the incidence of adverse childhood experiences
Source: Int J Public Health. 2019 May 28;64(7):1059–68. doi: 10.1007/s00038-019-01258-5 (PMC6677714; doi:10.1007/s00038-019-01258-5)
Supplement: Supplementary file 1 — Supplementary material 1 (DOCX 267 kb) [file 38_2019_1258_MOESM1_ESM.docx]

**Online-Only Supplement**

**Captions**

**eFigure1:** Direct Acyclic Graph (DAG) of the assumed associations between household income, community resources, covariates, and adverse childhood experience (ACE) incidence by age 8 years

**eMethods 1:** Adverse childhood experiences (ACEs) and effect modifier operationalization

**eMethods 2:** Estimation using inverse probability weights (IPW)

**eTable 1:** Inverse probability weighted risk differences in cumulative adverse childhood experience (ACE) incidence between households above and below the poverty line, for each of the seven measured ACEs, in the Growing Up in Scotland birth cohort study sample, followed from 10-months to 8 years (2004/2005-2013/2014, N=2,816)

**eTable 2:** Crude incidence proportions (1 or more adverse childhood experiences (ACEs), 3 or more ACEs) in joint strata of income and community resources, in the Growing Up in Scotland birth cohort study sample, followed from 10-months to 8 years (2004/2005-2013/2014, N=2,816)

**eTable 3:** Inverse probability weighted differences (RD) in cumulative incidence of 1 or more adverse childhood experiences (ACEs) by age 8 years across strata of income, park proximity, housing, transportation, breastfeeding education, and childcare services, and estimation of effect modification on the additive scale (departure from additivity), in the Growing Up in Scotland birth cohort study sample, followed from 10-months to 8 years (2004/2005-2013/2014, N=2,816)

**eTable 4:** Inverse probability weighted differences (RD) in cumulative incidence of 3 or more adverse childhood experiences (ACEs) by age 8 years across strata of income, park proximity, housing, transportation, breastfeeding education, and childcare services, and estimation of effect modification on the additive scale (departure from additivity), in the Growing Up in Scotland birth cohort study sample, followed from 10-months to 8 years (2004/2005-2013/2014, N=2,816)

**eTable 5:** Estimate of the maximum size of an unmeasured factor’s association, on the relative risk (RR) scale, with the mediator (RR=*γ*) and the outcome (RR= λ) for the true controlled direct estimates to be explained completely by unmeasured confounding (i.e. to be null, RR=1) despite non-null estimates (smallest combination encircled), for the Growing Up in Scotland birth cohort study sample, followed from 10-months to 8 years (2004/2005-2013/2014, N=2,816)

**eFigure 1:** Direct Acyclic Graph (DAG) of the assumed associations between household income, community resources, covariates, and adverse childhood experience (ACE) incidence by age 8 years


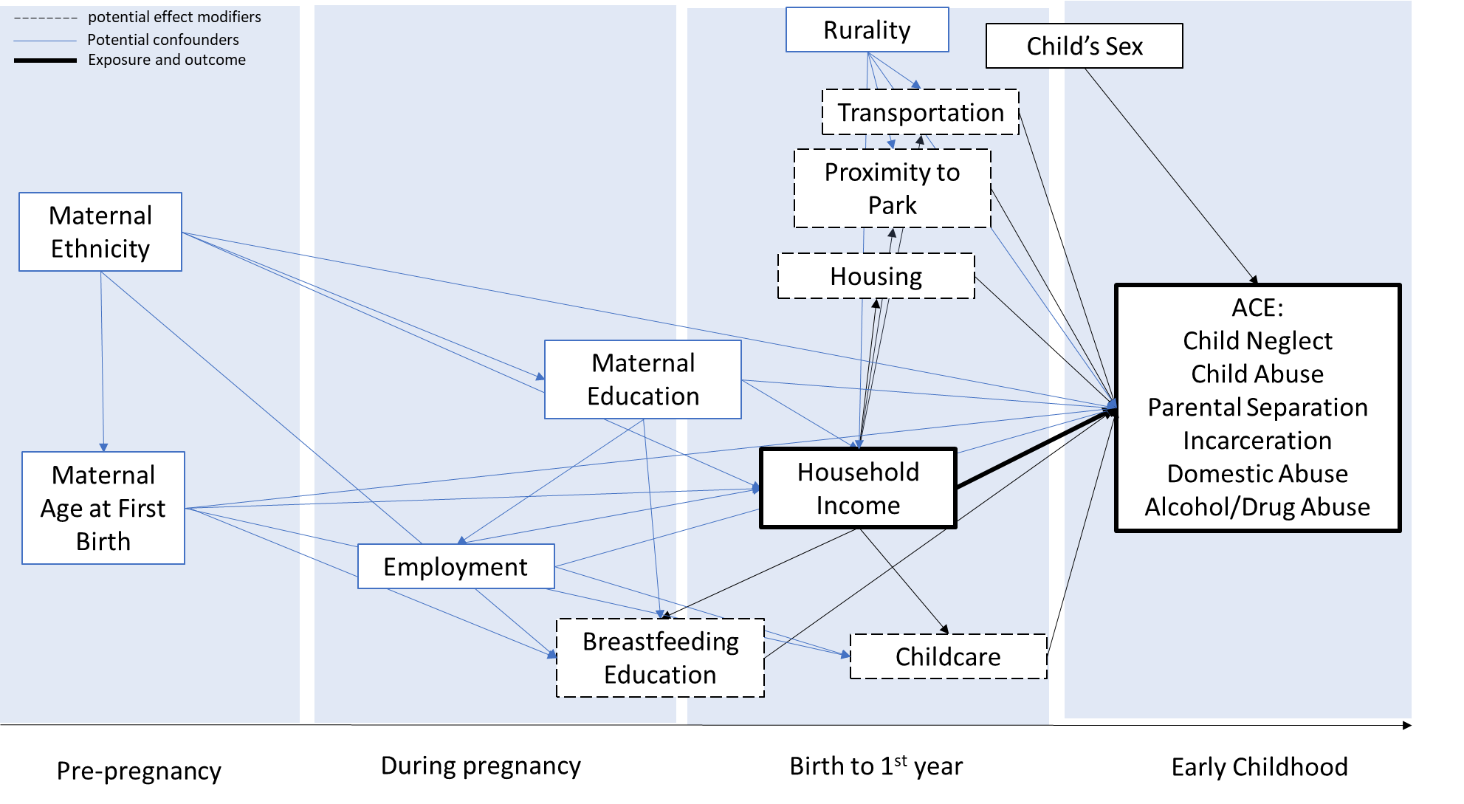


**eMethods 1**

**Adverse childhood experiences (ACEs)** **operationalization**

Proxies for these ACEs were measured in the Growing Up in Scotland (GUS) cohort as follows:

*Abuse*

Physical abuse, measured at sweep 7, was considered present if, to any of the statements “My parents smack me when I have done something wrong” and “You slap [child’s name] when they have done something wrong,” the child or their parent answered: “Often or always.” Domestic abuse and violence, measured at sweep 6, was considered present if the mother or stepmother of the child responded “yes” and “four or more times” to any of the following questions: “A since the child was born, has any partner or ex-partner ever pushed you or held you down?” or “Kicked, bitten or hit you?” or “Used a weapon against you?” or “choked or tried to strangle or smother you?” or “forced you or tried to force you to take part in any sexual activity when you did not want to.” Excluded from this domain is a proxy for the psychological abuse of the child (e.g. Was the child every sworn at, hit, put down, humiliated or made to fear for their physical safety?)—which was not asked by the GUS questionnaire. Similarly, though questions pertaining to sexual abuse were included in the GUS questionnaire, the number of children reporting it was too small for the data to be used (n<5), following the confidentiality agreement signed with the United Kingdom Data Service.

*Neglect*

Emotional neglect was considered present if the child answered “never” to any of the following statements: “My parents ask about my day in school,” “my parents play games or do other fun things with me,” “my parents help me with my homework,” “my parents tell me when I’m doing a good job with something,” or “my parents check to make sure I’m doing okay.” Excluded from this domain is a proxy for material neglect (e.g. Did the child ever feel there was not enough to eat, that there were only dirty clothes to wear, that there was no one to protect them). This item was also not covered in the GUS questionnaire.

*Alcohol and drug use*

Parental misuse of alcohol or drugs, measured at all sweeps except the 4^th^, was considered present if a parent answered “yes” to the question “[since the last interview] has the child experienced […] drug taking/alcoholism in the immediate family?” or if a parent reported any use of street drugs in the previous 12 months, or consumption of 14 or more units of alcohol per week (which has been identified as a hazardous level of alcohol consumption).^[[1]](#footnote-1)^

*Parental mental health*

Mental health problems were identified through three questionnaire items. First, parental mental health symptomatology was considered present if, at sweeps 6-7, a parent responded “yes” to the question “[since the last interview there was] mental disorder in the immediate family.” Second, in sweeps 2 and 4, respondents were asked to report their agreement with three items of the Depression component of the Depression, Anxiety and Stress (DASS) Depression scale^[[2]](#footnote-2)^: [In the past week] “I felt that I had nothing to look forward to;” “I felt sad and depressed;” and “I was unable to become enthusiastic about anything.” Scores for each item ranged from 0 [Did not apply to me at all] to 3 [applied to me very much or most of the time], leading to a sum of up to 9. DASS z-scores for each respondent were estimated, and respondents with scores over 1 standard deviation above the mean (z-score ≥ 1) were considered to have moderate to severe depressive symptomatology.^[[3]](#footnote-3)^ Thirdly, at sweeps 1, 3, and 5, respondents were administered questions from the SF-12 scale’s mental health component, which yields a general mental health score for the previous four weeks (Ware, Kosinski and Keller 1996). We used a previously-identified cut-off score of 36 to identify respondents with moderate to severe depressive symptomatology (Gill et al. 2007).

*Parental separation or incarceration*

Parents were considered separated or divorced if only one natural parent was present in the household at any of the seven sweeps. Parental incarceration was considered present if a parent answered “yes” to the question “Has your child experienced […] [having] a parent in prison [since the last interview].” This question was posed at sweeps 2, and 5-7.

*Summary ACE score*

Since ACE-related questionnaire items were asked inconsistently through the cycles of the study, we measured an 8-year cumulative incidence of ACEs using a summary score. ACE scores were estimated by summing the ACEs present for each child throughout the study period (range from 0 to 7). A binary variable of zero ACEs (versus one or more) was derived for the analysis of predictors of ACE absence (i.e. resilience to ACE incidence).

**eMethods 2**

**Associations between each resource and adverse childhood experience (ACE) incidence (Objective 1)**

To assess whether any of the identified community resources were protective against 8-year cumulative ACE incidence among both low-income and higher-income households (Objective 1), we estimated marginal risk differences (RD) in cumulative ACE incidence for each of the five resources using income-stratified, inverse probability-weighted (IPW) identity-link Poisson regression models.

These models were applied to obtain measures of association on the additivity scale (i.e. risk differences), as recommended for public health literature. Inverse probability weights for these analyses were estimated by first specifying a covariate-adjusted logistic regression for each resource, in each income strata. Predicted probabilities (propensity scores) for resource access were estimated using each of these models, and weights were estimated according to respondent’s observed resource access (i.e. weights of [1/propensity score] for those with the resource and [1/(1-propensity score)] for those without).

These weights ensured that within each income group, respondents were balanced in terms of the measured covariates (thereby minimizing confounding bias by these factors). Inverse probability weights were then multiplied with the respondents’ longitudinal survey weights (as is recommended for survey settings)^[[4]](#footnote-4)^. Growing Up in Scotland (GUS) longitudinal weights account for selection at baseline and non-response up until the last sweep of the study.^[[5]](#footnote-5)^

**Proportion of income inequalities that could be eliminated if all had the resources**

To assess the extent to which income inequalities in 8-year cumulative ACE incidence could be eliminated if access to identified resources were made equitable in the population (Objective 2), we estimated a metric known as the “Proportion Eliminated” (PE) in mediation and causal inference literature.^[[6]](#footnote-6)^ The Proportion Eliminated is estimated by first taking the difference between the total association between low income and ACE incidence (hereafter referred to as the “total effect” (TE) and the association between low income and ACE incidence that would remain if all had the mediating resource (hereafter referred to as the “controlled direct effect” (CDE). This difference is then divided by the total effect [i.e. PE= (TE-CDE)/TE].^[[7]](#footnote-7)^

*Total effect (TE) estimation*

To estimate the TE, an inverse probability weighted identity-link Poisson regression was specified for ACE incidence according to income. The inverse probability weights used in the latter model were estimated using propensity scores from a logistic model for low income regressed on covariates (IPW_income_). Weights were estimated according to respondent’s observed income (i.e. weights of [1/propensity score] for those with low income, and [1/(1-propensity score)] for those with income above the poverty line). These weights (IPW_income_) were then multiplied with longitudinal weights to form a final weight for low-income exposure. In this weighted model, the coefficient associated with low-income represents the population-average difference in cumulative ACE incidence association between groups above and below the poverty line (TE).

*Controlled direct effect (CDE) estimation*

To estimate the CDE, a similar weighted model was applied, this time adjusted for low income (RD_10_), resource absence (RD_01_), and the joint experience of low income and resource absence (RD_11_). In these CDE models, the coefficient associated with low-income and resource presence (RD_10_) gives an estimate of the controlled direct effect (CDE) of low-income when all have access to the resource.

Inverse probability weights used for CDE estimation were estimated by first specifying a logistic model for each resource, adjusted for all covariates and low income. Propensity scores from these models were used to estimate weights for the absence of each resource (IPW_resource_). Weights were estimated according to respondent’s observed resource access (i.e. weights of [1/propensity score] for those without the resource, and of [1/(1-propensity score)] for those with the resource).

Resource weights (IPW_resource_) were multiplied with income weights (IPW_income_) to create a final weight (IPW_income_*IPW_resource_ = IPW_income*resource_) that ensured all groups were balanced in terms of measured covariates. This final weight was also multiplied with respondents’ longitudinal weight, to account for selection at baseline and attrition.

*Confidence interval estimation*

All confidence intervals (95%) for the TE, CDE, and PE were estimated using the bootstrap method (500 iterations).

**eTable 1:** Inverse probability weighted^a^ risk differences in cumulative adverse childhood experience (ACE) incidence between households above and below the poverty line, for each of the seven measured ACEs, in the Growing Up in Scotland birth cohort study sample, followed from 10-months to 8 years (2004/2005-2013/2014, N=2,816)

| **Adverse childhood experience experienced by child by the age of 8 years** | **Cumulative incidence among lower-income households**  (%) | **Cumulative incidence among higher-income households**  (%) | **Difference in cumulative ACE incidence between households above and below the poverty line**  RD % (95% CI)^b^ |
| --- | --- | --- | --- |
| Child physical abuse | 25.7 | 19.2 | 5.1 (3.2, 7.1) |
| Child emotional neglect | 27.8 | 16.6 | 11.6 (9.7, 13.5) |
| Domestic violence | 1.5 | 6.8 | 4.7 (3.9, 5.4) |
| Household drug use, elevated alcohol use | 20.1 | 10.3 | 7.7 (6.1, 9.2) |
| Separation, divorce | 65.1 | 13.6 | 50.5 (48.2, 53.1) |
| Incarceration of parent | *n.a.*^c^ | *n.a.*^c^ | *n.a.*^c^ |
| Elevated mental health symptoms | 40.9 | 19.7 | 23.2 (21.0, 25.5) |

CI: Confidence interval. RD: Risk difference.

^a^  For low-income respondents, the minimum, mean and maximum of weights were [0.7, 1.55, 20.4], while for higher-income respondents they were [0.8, 9.3, 41.1].

^b^ Risk differences (RD) are expressed in percentage-point differences. RDs are weighted for balance according to child’s sex, mother’s age at birth of first child, mother’s visible minority status, maternal educational attainment, employment status at time of pregnancy, and rural of residence.

^c^ Proportions, and regression-based incidence differences not reported due to UK data service confidentiality restrictions (small number of respondents whose parents were incarcerated).

**eTable 2** Cumulative incidence of 1 or more adverse childhood experiences (ACEs), or of 3 or more ACEs by age 8 years, across strata of income and five community resources, in the Growing Up in Scotland birth cohort study sample, followed from 10-months to 8 years (2004/2005-2013/2014, N=2,816)

| **Community**  **resources** | **1 more ACEs by age 8 years** | | **3 or more ACEs by age 8 years** | |
| --- | --- | --- | --- | --- |
|  | **Above poverty line** (£11,000/year) (%) | **Below poverty line** (£11,000/year) (%) | **Above poverty line** (£11,000/year) (%) | **Below poverty line** (£11,000/year) (%) |
| **Overall** | 50.6 | 83.0 | 4.6 | 23.8 |
| **Housing** |  |  |  |  |
| Yes | 49.5 | 83.0 | 5.2 | 29.2 |
| No | 59.1 | 83.1 | 8.9 | 27.4 |
| **Walking proximity to park/playpark**^a^ |  |  |  |  |
| Yes | 54.5 | 89.2 | 6.1 | 30.9 |
| No | 50.4 | 88.9 | 5.2 | 21.9 |
| **Transportation** |  |  |  |  |
| Yes | 49.0 | 80.0 | 4.7 | 25.5 |
| No | 62.5 | 89.9 | 10.9 | 34.7 |
| **Breastfeeding education** |  |  |  |  |
| Yes | 48.8 | 82.0 | 5.3 | 26.8 |
| No | 56.3 | 86.3 | 6.8 | 33.2 |
| **Childcare** |  |  |  |  |
| Yes | 49.4 | 86.7 | 5.6 | 32.6 |
| No | 51.5 | 80.0 | 5.6 | 26.2 |

^a^ Park-related analyses were restricted to those living in urban settings.

**eTable 3:** Inverse probability weighted differences (RD) in cumulative incidence of 1 or more adverse childhood experiences (ACEs), by age 8 years across strata of income, park proximity, housing, transportation, breastfeeding education, and childcare services, and estimation of effect modification on the additive scale (departure from additivity), in the Growing Up in Scotland birth cohort study sample, followed from 10-months to 8 years (2004/2005-2013/2014, N=2,816)

|  | **Income strata** | | | | | **Effect modification on additive scale**  if  [RD_11_-RD_10_-RD_01_] ≠ 0 (95% CI)^b^ |
| --- | --- | --- | --- | --- | --- | --- |
|  | | **Above poverty line** | | **Below poverty line** | |  |
| **Resource strata** | | N with/N without ≥1 ACEs  (IPW-weighted incidence proportion, %) | ≥ 1 ACEs  RD %  ^a^ (95%CI) | N with/N without ≥1 ACEs  (IPW-weighted incidence proportion, %) | ≥1 ACEs  RD % ^a^ (95%CI) |  |
| **Housing** | |  | |  | |  |
| No | | 146/101 (59%) | 15.5 (13.3, 17.8) (RD_01_) | 123/23 (83%) | 37.2 (35.4, 38.9) (RD_11_) | -1.5 (-18.0, -13.0) |
| Yes | | 983/1002 (50%) | 0 (reference) (RD_00_) | 362/74 (83%) | 37.1 (35.3, 39.0) (RD_10_) |  |
| **Park proximity^c^** | |  | |  | |  |
| No | | 62/66 (48%) | -8.4 (-10.9, -5.6) | 33/6 (85%) | 36.1 (34.1, 38.1) | 10.5 (7.7, 13.3) |
| Yes | | 780/726 (52%) | 0 (reference) | 366/60 (86%) | 33.9 (31.9, 36.0) |  |
| **Transportation** | |  | |  | |  |
| No | | 167/100 (63%) | 12.6 (10.2,15.0) | 160/18 (90%) | 40.5 (38.8, 42.2) | -4.8 (-7.5, -2.2) |
| Yes | | 962/1003 (49%) | 0 (reference) | 325/81 (80%) | 32.8 (30.8, 34.7) |  |
| **Breastfeeding Education** | |  | |  | |  |
| No | | 285/221 (56%) | 7.5 (5.2, 9.9) | 157/25 (86%) | 40.5 (38.8, 42.3) | -2.5 (-5.1, 0.0) |
| Yes | | 835/875 (49%) | 0 (reference) | 327/72 (82%) | 35.6 (33.7, 37.5) |  |
| **Childcare** | |  | |  | |  |
| No | | 392/369 (52%) | 1.9 (-0.4, 4.2) | 252/63 (80%) | 28.6 (26.8, 30.4) | -10.3 (-12.9, -7.7) |
| Yes | | 655/672 (49%) | 0 (reference) | 208/32 (87%) | 37.0 (35.2, 38.8) |  |

NOTE: RD= cumulative incidence risk differences, CI=Confidence Interval.

^a^ RDs are adjusted for mother or stepmother’s ethnicity, age at birth of the child, employment status at during pregnancy, area of residence, level of educational attainment, and child’s sex. ^b^ Confidence intervals are obtained via the Delta method. ^c^ Park-related analyses were restricted to those living in urban settings.

**eTable 4:** Inverse probability weighted differences (RD) in cumulative incidence of 3 or more adverse childhood experiences (ACEs), by age 8 years across strata of income, park proximity, housing, transportation, breastfeeding education, and childcare services, and estimation of effect modification on the additive scale (departure from additivity), in the Growing Up in Scotland birth cohort study sample, followed from 10-months to 8 years (2004/2005-2013/2014, N=2,816)

|  | **Income strata** | | | | | **Effect modification on additive scale**  if  [RD_11_-RD_10_-RD_01_] ≠ 0 (95% CI)^b^ |
| --- | --- | --- | --- | --- | --- | --- |
|  | | **Above poverty line** | | **Below poverty line** | |  |
| **Resource strata** | | N with/N without ≥3 ACEs  (IPW-weighted incidence proportion, %) | ≥3 ACEs  RD %  ^a^ (95%CI) | N with/N without ≥3 ACEs  (IPW-weighted incidence proportion, %) | ≥3 ACEs  RD % ^a^ (95%CI) |  |
| **Housing** | |  | |  | |  |
| No | | 18/229 (7%) | 4.2 (3.0, 5.3) (RD_01_) | 35/113 (24%) | 23.2 (22.1, 24.2) (RD_11_) | -3.9 (-5.8, -2.1) |
| Yes | | 85/1900 (4%) | 0 (reference) (RD_00_) | 104/332 (24%) | 22.9 (21.5, 24.4) (RD_10_) |  |
| **Park proximity^c^** | |  | |  | |  |
| No | | 6/122 (5%) | -1.3 (-2.4, 0.0) | 8/31 (21%) | 22.7 (21.2, 24.3) | -2.1 (-4.3, 0.0) |
| Yes | | 75/1431 (5%) | 0 (reference) | 111/315 (26%) | 26.2 (24.6, 27.8) |  |
| **Transportation** | |  | |  | |  |
| No | | 22/245 (8%) | 3.5 (2.3, 4.8) | 54/124 (30%) | 15.4 (10.9, 20.7) | 11.4 (9.5, 13.3) |
| Yes | | 81/1884 (4%) | 0 (reference) | 85/321 (21%) | 18.0 (16.6, 19.4) |  |
| **Breastfeeding Education** | |  | |  | |  |
| No | | 30/476 (6%) | 2.2 (1.0, 3.4) | 48/138 (26%) | 27.3 (25.9, 28.6) | 4.5 (2.4, 6.4) |
| Yes | | 73/1637 (4%) | 0 (reference) | 91/308 (23%) | 20.6 (19.2, 22.1) |  |
| **Childcare** | |  | |  | |  |
| No | | 34/727 (5%) | 1.0 (0.0,2.0) | 72/243 (23%) | 20.4 (19.2, 21.5) | -8.2 (-10.1, -6.2) |
| Yes | | 61/1266 (5%) | 0 (reference) | 62/172 (26%) | 27.5 (26.0, 29.0) |  |

NOTE: RD= cumulative incidence risk differences, CI=Confidence Interval.

^a^ RDs are adjusted for mother or stepmother’s ethnicity, age at birth of the child, employment status at during pregnancy, area of residence, level of educational attainment, and child’s sex. ^b^ Confidence intervals are obtained via the Delta method. ^c^ Park-related analyses were restricted to those living in urban settings.

**eTable 5** Estimate of the maximum size of an unmeasured factor’s association, on the relative risk scale (RR), with the mediator (RR=*γ*) and the outcome (RR= λ) for the true controlled direct estimates (CDE) to be explained completely by unmeasured confounding (i.e. to be null, RR=1) despite non-null estimates (smallest combination encircled), in the Growing Up in Scotland birth cohort study sample, followed from 10-months to 8 years (2004/2005-2013/2014, N=2,816)

|  | Expected CDE of low income on cumulative incidence (≥ 1 ACEs)  when observed CDE if all had housing (PR = 1.73) is divided by bounding formula *((γ λ) / (γ + λ – 1))* ^a^ | | | | | | | | | | | | |
| --- | --- | --- | --- | --- | --- | --- | --- | --- | --- | --- | --- | --- | --- |
|  | ***γ*** | | | | | | | | | | | | |
| ***λ*** | **1.01** | **1.05** | **1.1** | **1.2** | **1.3** | **1.4** | **1.5** | **2** | **2.5** | **3** | **4** | **5** |  |
| **1.01** | 1.73 | 1.73 | 1.73 | 1.73 | 1.73 | 1.73 | 1.72 | 1.72 | 1.72 | 1.72 | 1.72 | 1.72 |  |
| **1.05** | 1.73 | 1.73 | 1.72 | 1.72 | 1.71 | 1.71 | 1.70 | 1.69 | 1.68 | 1.68 | 1.67 | 1.66 |  |
| **1.1** | 1.73 | 1.72 | 1.72 | 1.70 | 1.69 | 1.69 | 1.68 | 1.65 | 1.64 | 1.63 | 1.61 | 1.60 |  |
| **1.2** | 1.73 | 1.72 | 1.70 | 1.68 | 1.66 | 1.65 | 1.63 | 1.59 | 1.56 | 1.54 | 1.51 | 1.50 |  |
| **1.3** | 1.73 | 1.71 | 1.69 | 1.66 | 1.64 | 1.62 | 1.60 | 1.53 | 1.49 | 1.46 | 1.43 | 1.41 |  |
| **1.4** | 1.73 | 1.71 | 1.69 | 1.65 | 1.62 | 1.59 | 1.57 | 1.48 | 1.43 | 1.40 | 1.36 | 1.33 |  |
| **1.5** | 1.72 | 1.70 | 1.68 | 1.63 | 1.60 | 1.57 | 1.54 | 1.44 | 1.38 | 1.35 | 1.30 | 1.27 |  |
| **2** | 1.72 | 1.69 | 1.65 | 1.59 | 1.53 | 1.48 | 1.44 | 1.30 | 1.21 | 1.15 | 1.08 | 1.04 |  |
| **2.5** | 1.72 | 1.68 | 1.64 | 1.56 | 1.49 | 1.43 | 1.38 | 1.21 | 1.11 | 1.04 | 0.95 | 0.90 |  |
| **3** | 1.72 | 1.68 | 1.63 | 1.54 | 1.46 | 1.40 | 1.35 | 1.15 | 1.04 | 0.96 | 0.87 | 0.81 |  |
| **4** | 1.72 | 1.67 | 1.61 | 1.51 | 1.43 | 1.36 | 1.30 | 1.08 | 0.95 | 0.87 | 0.76 | 0.69 |  |
| **5** | 1.72 | 1.66 | 1.60 | 1.50 | 1.41 | 1.33 | 1.27 | 1.04 | 0.90 | 0.81 | 0.69 | 0.62 |  |
|  | Expected CDE of low income on cumulative incidence (≥ 3 ACEs)  when observed CDE if all had transportation (PR = 1.66) is divided by bounding formula *((γ λ) / (γ + λ – 1))* ^a^ | | | | | | | | | | | |  |
| **1.01** | 1.66 | 1.66 | 1.66 | 1.66 | 1.66 | 1.66 | 1.65 | 1.65 | 1.65 | 1.65 | 1.65 | 1.65 |  |
| **1.05** | 1.66 | 1.66 | 1.65 | 1.65 | 1.64 | 1.64 | 1.63 | 1.62 | 1.61 | 1.61 | 1.60 | 1.60 |  |
| **1.1** | 1.66 | 1.65 | 1.65 | 1.63 | 1.63 | 1.62 | 1.61 | 1.58 | 1.57 | 1.56 | 1.55 | 1.54 |  |
| **1.2** | 1.66 | 1.65 | 1.63 | 1.61 | 1.60 | 1.58 | 1.57 | 1.52 | 1.49 | 1.48 | 1.45 | 1.44 |  |
| **1.3** | 1.66 | 1.64 | 1.63 | 1.60 | 1.57 | 1.55 | 1.53 | 1.47 | 1.43 | 1.40 | 1.37 | 1.35 |  |
| **1.4** | 1.66 | 1.64 | 1.62 | 1.58 | 1.55 | 1.52 | 1.50 | 1.42 | 1.38 | 1.34 | 1.30 | 1.28 |  |
| **1.5** | 1.65 | 1.63 | 1.61 | 1.57 | 1.53 | 1.50 | 1.48 | 1.38 | 1.33 | 1.29 | 1.25 | 1.22 |  |
| **2** | 1.65 | 1.62 | 1.58 | 1.52 | 1.47 | 1.42 | 1.38 | 1.25 | 1.16 | 1.11 | 1.04 | 1.00 |  |
| **2.5** | 1.65 | 1.61 | 1.57 | 1.49 | 1.43 | 1.38 | 1.33 | 1.16 | 1.06 | 1.00 | 0.91 | 0.86 |  |
| **3** | 1.65 | 1.61 | 1.56 | 1.48 | 1.40 | 1.34 | 1.29 | 1.11 | 1.00 | 0.92 | 0.83 | 0.77 |  |
| **4** | 1.65 | 1.60 | 1.55 | 1.45 | 1.37 | 1.30 | 1.25 | 1.04 | 0.91 | 0.83 | 0.73 | 0.66 |  |
| **5** | 1.65 | 1.60 | 1.54 | 1.44 | 1.35 | 1.28 | 1.22 | 1.00 | 0.86 | 0.77 | 0.66 | 0.60 |  |

^a^ Formula described in: VanderWeele, T.J., 2016. Mediation Analysis: A Practitioner's Guide. Annual Review of Public Health 37:17-32.

**eTable 6** Description of propensity scores for access to housing, a park or playpark, transportation, childcare, and breastfeeding education, in strata of income, for the Growing Up in Scotland birth cohort study sample, followed from 10-months to 8 years (2004/2005-2013/2014, N=2,816)

|  | **Below poverty line** | | **Above poverty line** | |
| --- | --- | --- | --- | --- |
|  | **Propensity scores**  (Minimum, Mean, Maximum) | **Inverse probability weights (IPW)**^a^  (Minimum, Mean, Maximum) | **Propensity scores**  (Minimum, Mean, Maximum) | **Inverse probability weights (IPW)** ^a^ (Minimum, Mean, Maximum) |
| **Housing** |  |  |  |  |
| No | 0.18, 0.32, 0.53 | 1.3, 6.4, 18.3 | 0.05, 0.16, 0.37 | 2.1, 9.9, 28.3 |
| Yes | 0.18, 0.25, 0.53 | 0.8, 2.0, 4.84 | 0.05, 0.11, 0.41 | 0.7, 1.0, 3.3 |
| **Park proximity**^b^ |  |  |  |  |
| No | 0.05, 0.10, 0.16 | 6.3, 2.9, 48.4 | 0.05, 0.08, 0.12 | 6.1, 11.8, 31.6 |
| Yes | 0.05, 0.09, 0.16 | 0.7, 1.7, 4.2 | 0.04, 0.08, 0.11 | 0.7, 1.0, 2.9 |
| **Transportation** |  |  |  |  |
| Yes | 0.20, 0.35, 0.49 | 1.8, 4.4, 10.1 | 0.07, 0.16, 0.45 | 1.9, 7.3, 16.8 |
| No | 0.20, 0.32,0.49 | 0.8, 2.3, 7.4 | 0.07, 0.13, 0.45 | 0.6, 1.0, 4.1 |
| **Breastfeeding education** |  |  |  |  |
| Yes | 0.16, 0.33, 0.51 | 1.5, 4.8, 12.3 | 0.08, 0.24, 0.38 | 1.8, 3.8, 17.1 |
| No | 0.16, 0.31, 0.51 | 0.9, 2.1, 6.0 | 0.08, 0.23, 0.38 | 0.8, 1.1, 3.7 |
| **Perceive adequate access to childcare** |  |  |  |  |
| Yes | 0.36, 0.59, 0.88 | 0.8, 2.8, 9.8 | 0.22, 0.42, 0.87 | 0.8, 2.7, 8.2 |
| No | 0.36, 0.51, 0.88 | 1.1, 3.8, 27.6 | 0.22, 0.33, 0.84 | 0.9, 1.5, 10.8 |

^a^ IPW weights represent model-based IPW weights that were multiplied with the sample’s longitudinal weight.

^b^ Park-related analyses were restricted to those living in urban settings.

**eTable 7:** Description of propensity scores for income and access to housing, a park or playpark, transportation, childcare, and breastfeeding education, and of inverse probability weights across strata of income and resource access, for total effect, controlled direct effect, and proportion eliminated analyses, for the Growing Up in Scotland birth cohort study sample, followed from 10-months to 8 years (2004/2005-2013/2014, N=2,816)

|  | **Propensity scores**  (Minimum, Mean, Maximum) | **Inverse probability**  **weights (IPW)^a^**  (Minimum, Mean, Maximum) |
| --- | --- | --- |
| **Income (all)** |  |  |
| Above poverty line | 0.08, 0.19, 0.92 | 0.6, 1.5, 23.3 |
| Below poverty line | 0.08, 0.46, 0.96 | 0.8, 9.2, 41.5 |
| **Income (urban only)** |  |  |
| Above poverty line | 0.11, 0.21, 0.93 | 0.7, 1.8, 35.8 |
| Below poverty line | 0.11, 0.51, 0.96 | 0.8, 9.6, 36.0 |
|  |  |  |
| **Housing** |  |  |
| No | 0.06, 0.14, 0.56 | 0.7, 3.5, 45.3 |
| Yes | 0.06, 0.22, 0.56 | 1.6, 44.8, 402.3 |
| **Park proximity**^b^ |  |  |
| No | 0.04, 0.08,0.14 | 0.8, 3.9, 39.4 |
| Yes | 0.05, 0.09, 0.14 | 7.0, 48.4, 295.0 |
| **Perceived adequate access to transportation** |  |  |
| Yes | 0.06, 0.16, 0.52 | 0.7, 3.44, 41.1 |
| No | 0.06, 0.23, 0.52 | 1.4, 37.8, 303.6 |
| **Breastfeeding education** |  |  |
| Yes | 0.11, 0.25, 0.43 | 0.9, 4.0, 55.0 |
| No | 0.11, 0.26, 0.43 | 1.9, 15.0, 238.3 |
| **Perceive adequate access to childcare** |  |  |
| Yes | 0.26, 0.36, 0.88 | 0.9, 5.3, 137.0 |
| No | 0.26, 0.46, 0.88 | 1.0, 10.3, 138.8 |

^a^ IPW weights represent model-based IPW weights that were multiplied with the sample’s longitudinal weight.

^b^ Park-related analyses were restricted to those living in urban settings.

1. Brown L, Campbell-Jack D, Gray L, et al. The Scottish Health Survey: Main Report. Edinburgh, UK: Scottish Government;2015. [↑](#footnote-ref-1)
2. Lovibond PF, Lovibond SH. The structure of negative emotional states: Comparison of the Depression Anxiety Stress Scales (DASS) with the Beck Depression and Anxiety Inventories. Behaviour Research and Therapy. 1995;33(3):335-343; Bradshaw P, Tipping S, Marryat L, Corbett J. *Growing Up In Scotland Sweep 2: 2006-2007 User Guide* Essex, UK: UK Data Archive;2007. [↑](#footnote-ref-2)
3. Parkitny L, McAuley J. The depression anxiety stress scale (DASS). *Journal of Physiotherapy.* 2010;56(3):204. [↑](#footnote-ref-3)
4. Nguyen QC, Osypuk TL, Schmidt NM, Glymour MM, Tchetgen Tchetgen EJ. Practical guidance for conducting mediation analysis with multiple mediators using inverse odds ratio weighting. *American journal of epidemiology.* 2015;181(5):349-356. [↑](#footnote-ref-4)
5. Anderson S, Bradshaw P, Cunningham-Burley S, et al. *Growing Up in Scotland: A study following the lives of Scotland's children.* Edinburgh: ScotCen2007. [↑](#footnote-ref-5)
6. VanderWeele T. *Explanation in causal inference: methods for mediation and interaction.* Oxford University Press; 2015. [↑](#footnote-ref-6)
7. VanderWeele TJ. Policy-relevant proportions for direct effects. *Epidemiology (Cambridge, Mass.).* 2013;24(1):175-176. [↑](#footnote-ref-7)
